# Biomimetic biphasic scaffolds for osteochondral defect repair

**DOI:** 10.1093/rb/rbv015

**Published:** 2015-08-24

**Authors:** Xuezhou Li, Jianxun Ding, Jincheng Wang, Xiuli Zhuang, Xuesi Chen

**Affiliations:** ^1^Key Laboratory of Polymer Ecomaterials, Changchun Institute of Applied Chemistry, Chinese Academy of Sciences, Changchun 130022, People’s Republic of China;; ^2^Department of Orthopedics, The Second Hospital of Jilin University, Changchun 130041, People’s Republic of China

**Keywords:** biomaterial, biomimetic, biphasic scaffold, osteochondral regeneration, tissue engineering

## Abstract

The osteochondral defects caused by vigorous trauma or physical disease are difficult to be managed. Tissue engineering provides a possible option to regenerate the damaged osteochondral tissues. For osteochondral reconstruction, one intact scaffold should be considered to support the regeneration of both cartilage and subchondral bone. Therefore, the biphasic scaffolds with the mimic structures of osteochondral tissues have been developed to close this chasm. A variety of biomimetic bilayer scaffolds fabricated from natural or synthetic polymers, or the ones loading with growth factors, cells, or both of them make great progresses in osteochondral defect repair. In this review, the preparation and *in vitro* and/or *in vivo* verification of bioinspired biphasic scaffolds are summarized and discussed, as well as the prospect is predicted.

5th China-Europe Symposium on Biomaterials in Regenerative Medicine (CESB 2015) Hangzhou, China April 7–10, 2015

## Introduction

**Figure 1.**
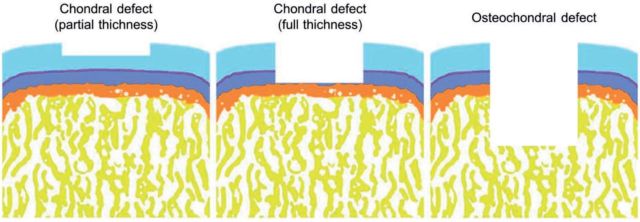
Classification of articular cartilage defects. Osteochondral defects characterize of the damage extending deep into the subchondral bone. (Reprinted with permission from Ref. [4])

**Figure 2.**
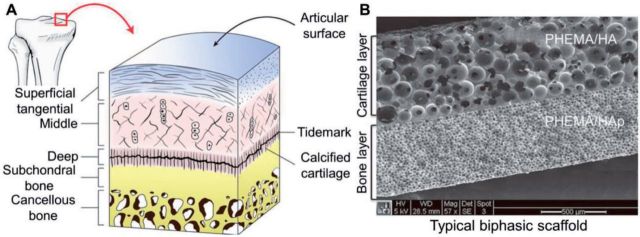
Typical histological structure of osteochondral unit (A). Typical scanning electron microscope (SEM) cross-section microimage of integrated bi-layered poly(2-hydroxyethyl methacrylate (PHEMA)/HA//PHEMA/HAp scaffold (B). (Reprinted with permission from Refs. [5, 32])

Cartilage regeneration as one of the most important orthopedic research areas has been intensively explored for decades [1]. Severe cartilage trauma often combines with the destruction of subchondral bone [2]. Besides, subchondral bone involving cartilage defects also can be caused by some physical diseases, such as osteochondritis dissecans (OCD) [3]. This kind of articular cartilage defects extending deeply into the subchondral bone is known as osteochondral defects (Fig. 1) [4]. In the original period, the reconstruction of osteochondral defects was focused on the upper layer of cartilage without consideration of lower subchondral tissue, so most of the repair results were disappointing (Fig. 1) [1]. Recently, the depth studies about the detail structures of osteochondral tissues bring researchers new inspiration about effectively regenerating osteochondral defects. As depicted in Fig. 2A, the osteochondral tissue structures can be divided into two major parts, including the upper zonal cartilage and the underlying subchondral bone, which possess different sub-structures and mechanical properties. The preparation of biomimetic scaffolds should follow the natural structures and aim at structurally integrating the osteochondral tissues.

### Structural features of osteochondral tissues

As shown in Fig. 2, the zonal cartilage layer consists of the superficial, middle, deep, and calcified cartilage zones [5]. The superficial zone is assembled by densely packed collagen type II (Col II) fiber paralleling to the joint surface, that is why it is strong in tension to the resistance of shear force on the surface [6]. The middle zone profits from arch shaped and obliquely oriented Col II fibril, an abundance of proteoglycans, and a few of cells, which possesses the main function of cushioning effect in vigorous exercise [7]. In the deep cartilage zone, the Col II fiber is tightly packed perpendicularly to the cartilage surface. In addition, it contains less water and more active cells, which proved more compressive strain for weight bearing [8]. Finally, the calcified cartilage mainly composes of calcified chondrocytes, responsibly for firmly anchoring the whole cartilage layer to the underlying subchondral bone [9].

Subchondral bone plate and cancellous bone form subchondral bone, which mainly contains of collagen type I (Col I), hydroxyapatite (HAp), and water. The subchondral bone provides support for upper cartilage layer. According to its composition and structure, it possesses more stiffness and compressive strength comparing to calcified cartilage [10]. The osteochondral defects are characterized by the deep cartilage damage to subchondral bone, so it is important to figure out the exact ingredients and their interaction with subchondral bone.

The interface as a connection of the upper cartilage and underlying subchondral bone is a complex of above two [11]. Structurally, the calcified cartilage is interdigitated with the subchondral bone plate. The vertically orientated Col fibrils extend from deep zone to calcified cartilage through a wavy tidemark, but does not enter into the subchondral bone. The vertically orientated Col fibrils just like micro-springs, which can absorb and spread weight bearing pressures to subchondral bone.

### Situation of osteochondral regeneration

In clinic, the commonly used methods to treat osteochondral defects include debridement and bone marrow stimulation technique, osteochondral grafts, etc. Debridement and bone marrow stimulation may not provide a satisfied long-term prognosis, especially in young active patients [12]. Although osteochondral grafts demonstrate satisfactory outcomes, the allografts face a limited application ascribed to immune rejection and the risk of disease transmission [13, 14], and the autografts will cause additional physical trauma [15, 16]. Therefore, there need alternative therapies for osteochondral defects urgently.

Tissue engineering always provides possible methods for tissue regeneration [17, 18], which has been applied in the reconstruction of many tissues and organs. For the repair of cartilage defects, the biodegradable scaffolds without or with growth factors and/or cells have been well employed [19–21]. Currently, almost all of the scaffolds from natural and/or synthetic polymers are homogeneous for simple cartilage defect repair. However, the traditional homogeneous scaffolds cannot balance chondrogenesis and osteogenesis simultaneously for repairing osteochondral defects. Thus the biphasic scaffolds characterized with different mechanical strengths and spatial structures of different parts, and even different loading abilities of growth factors are required to meet the demands. The upper layer supports chondrogenesis for cartilage regeneration, and the underlying part serves as a template for osteogenesis in the repair of subchondral bone.

Besides the abiotic factors, like inorganic scaffolds themselves, the biotic factors, such as growth factors and cells, also play important roles in the reconstruction of osteochondral defects [22]. As aforementioned, growth factors have been proved exhibited a pivotal position in tissue regeneration, which have been well investigated in regenerating simple cartilage defects [23]. As a typical instance, transforming growth factor-β1 (TGF-β1) loaded in various scaffolds can promote the cartilage regeneration through promoting the initial stage of mesenchymal condensation, prechondrocyte proliferation, and production of extracellular matrix and cartilage-specific molecule deposition [24]. There is no exception that growth factors in biphasic scaffolds serve as important roles as those in homogenous scaffolds. Sometimes biphasic scaffolds just load one kind of growth factors in a specific layer to facilitate cartilage or bone regeneration [25]. In order to embody the advantages of biphasic scaffolds with a well-designed interface, both TGF and bone morphogenetic protein-2 (BMP-2) are enveloped into different parts simultaneously [26]. In addition, the cells mostly used in cartilage tissue engineering are chondrocytes and mesenchymal stem cells (MSCs). Most of *in vivo* animal and clinical studies demonstrated the positive results of scaffolds with transplanted cells compared with those of cell-free ones [27–30].

This review focuses on summarizing the fabrication of biphasic scaffolds for the repair of osteochondral defects, presenting current challenges, as well as predicting the future directions.

## BIOMIMETIC BILAYER SCAFFOLDS FOR OSTEOCHONDRAL REGENERATION

An increasing number of advanced scaffolds have been preclinically determined toward osteochondral defect animal models, and most of them are biphasic [31]. Different materials have been explored in the synthesis processes of these bioinspired biphasic scaffolds, which possess various properties. As mentioned before, similarly to their complexities and natural structures of osteochondral tissues, the biphasic scaffolds are prepared with two parts: cartilage segment and subchondral moiety (Fig. 2B) [32]. Usually, the upper cartilaginous layer composes of lower strength hydrogels [33], etc., and underlying subchondral layer consists of higher strength scaffolds, such as, tricalcium phosphate (TCP) [34] and bioceramics [35].

### Components of partial scaffolds for cartilage repair

The natural polymers possess more favorable biocompatibility, but less controllable compare to the synthetic ones. The natural material-originated scaffolds may not provide high mechanical strength as the scaffolds from synthetic polymers, whereas the weight bearing can be controlled post-operation in clinic. Therefore, high mechanical strength does not necessary at the primary stage [36]. Hydrogels made of natural or synthetic hydrophilic polymers are most commonly used to regenerate the chondral layer of joint. The natural materials, including fibrin [37], hyaluronan (HA) [38, 39], Col [40–43], chitosan [44], alginate [2, 26], silk fibroin [45], and their compounds have been most widely applied to support cartilage repair in a wide range of osteochondral scaffolds.

In addition, the synthetic polymers, such as polylactide (PLA), polyglycolide, poly(lactide-*co*-glycolide) (PLGA), and poly(ε-caprolactone) (PCL), can be fabricated into various scaffolds with different mechanical properties and degradation rates, which have been used in both chondral and subchondral bone layers [27, 46–48]. Moreover, the scaffolds can be fabricated into various shapes with desired porosity. Although they are more controllable and easy to be handled as we all know, there are still some limitations of synthetic materials, including poor cell adhesion. Fortunately, the poor cell attachment can be diminished by surface disposing or mixing some natural materials, like chondroitin sulfate [49], silicate [26], and chitosan [39].

### Ingredients of partial scaffolds for subchondral bone regeneration

Like other orthopedic implants, the scaffolds for subchondral bone regeneration should possess excellent biocompatibility and biodegradability, suitable mechanical strength similar to cancellous bone and good bone ingrowth.

The biocompatible and biodegradable ceramic materials, including HAp, TCP, and so on, have been widely used. They can provide similar mechanical property as cancellous bone in the early stage, and can be further completely replaced by natural sponge bone. As reported previously, TCP alone or in combination with PCL, Col, or HAp all can improve the subchondral bone’s regeneration [40, 46–48, 50, 51]. Bioglasses and metallic materials have also been used in the repair of subchondral bone. The bioglasses combining with PLGA as subchondral bone scaffolds yield the best histological score, but play a critical role in the spongy bone's reconstruction [49]. Titanium and porous tantalum implants can achieve excellent subchondral bone integration and good histological score results [52, 53]. Besides the above high stiffness materials, the synthetic polymers, such as PLA, PLGA, PCL, poly(2-hydroxyethyl methacrylate) (PHEMA), alone or combined with natural materials, also have been employed as promising matrices in the subchondral bone's regeneration [26, 27, 32, 37, 39, 47, 53–55].

## PRECLINICAL EVALUATION OF BIPHASIC SCAFFOLDS

Biphasic scaffolds have been assessed *in vitro* and toward osteochondral defect animal models *in vivo*. Different strategies are applied and evaluated, such as implantation of bare scaffolds or the ones seeded with chondrocytes or MSCs and encapsulated growth factors.

### Bare biphasic scaffolds in osteochondral defect reconstruction

Some of biphasic scaffolds are directly implanted into the local osteochondral defect region without loading any growth factors or cells, although biotic factors are considered as important parts in tissue engineering.

For example, Frenkel *et al.* [39] used the biphasic scaffolds consisting of a polyelectrolytic complex (PEC) hydrogel of HA and chitosan or a Col I scaffold as cartilaginous layer, and poly(D,L-lactide) (PDLLA) invested with HAp as osteogenic layer to repair the rabbit’s osteochondral defects without any biotic factors. Twenty four weeks later, both the scaffolds completely degraded, and the osteochondral defects were well repaired. In detail, the implantation of scaffold with Col I in cartilage layer created the highest percentage of hyaline-appearing cartilage in the repair, while the PEC-incorporated scaffold produced the greatest bonding degree of repair to the host, structural integrity of neocartilage, and reconstitution of subchondral bone.

**Figure 3.**
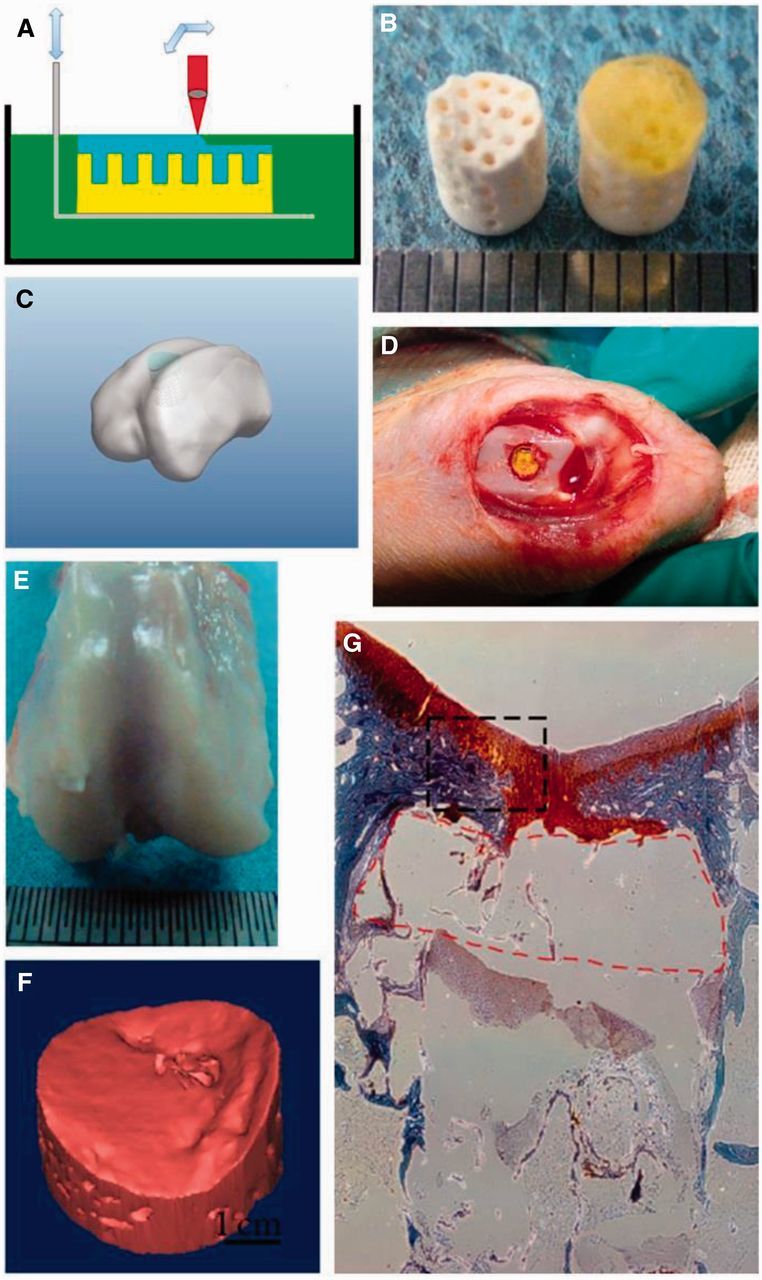
Schematic illustration of integration of chondral phase and osseous part *via* stereolithography (A). Fabricated ceramic scaffold (Left) and PEG/β-TCP scaffold (Right; B). The cured PEG hydrogel is tightly anchored to the underlying ceramic substrate. Illustration of scaffold implantation in rabbit trochlea osteochondral defects (C and D). Gross appearance of repaired cartilage (E), 3D model of repaired subchondral bone (F), and histology of repaired cartilage (G) after implantation of PEG/β-TCP scaffold for 52 weeks. (Reprinted with permission from Refs. [34, 57])

Three-dimensional (3D) printing biphasic scaffolds have been first reported in 2002 [56]. Sherwood *et al.* [56] developed the unique, heterogeneous, and osteochondral scaffolds by 3D printing process. The upper cartilage region was composed of poly(D,L-lactide-*co*-glycolide) and poly(L-lactide) with a porosity of 90%, and the lower cloverleaf-shaped bone portion was 55% porous and consisted of a poly(L-lactide-*co*-glycolide)/TCP composite. The transition region between these two sections contained a gradient of materials and porosity to prevent delamination. Chondrocytes preferentially attached to the cartilage portion of the device, and cartilage formed during a 6-week *in vitro* culture period. The tensile strength of bone region was similar in magnitude to fresh human cancellous bone. The declared advantages indicated the great potential of 3D printing heterogeneous scaffold in clinical regeneration of osteochondral defects. Zhang *et al.* [34] also fabricated a biphasic poly(ethylene glycol) (PEG)/β-TCP scaffold with enhanced interfacial integration through 3D printing technique (Fig. 3). The PEG hydrogel as chondral phase was directly cured on the interface of β-TCP (*i.e.,* osseous phase) layer by layer to fabricate osteochondral scaffolds. The biomimetic scaffolds with interface structure enhanced the integration of osteochondral tissues. After one year implantation in rabbit trochlea osteochondral defect model, the hyaline-like cartilage formed along with white smooth surface and typical tidemark appeared at 52 weeks, and the subchondral bone was repaired in a ‘flow like’ manner from surrounding bone to the defect center (Fig. 3) [57]. The results implied that the biphasic PEG/*β*-TCP composites fabricated by 3D printing provided a feasible strategy for osteochondral tissue reconstruction.

Besides, Sosio *et al.* [58] compared the 3D bicomponent substitutes made of Col I and HAp without and with seeding autologous chondrocytes in four pigs. The histologic evaluation showed the quality of reparative tissues seemed superior for the lesions with the unseeded scaffolds. Several other studies also indicated that there were no differences in healing of the defects for implant with addition/omission of autologous costochondral chondrocytes [49, 58] or even better with the unseeded scaffolds. Of course, the relatively negative results did not deny the role of biotic factors in the reconstruction of osteochondral defect.

### Biphasic scaffolds encapsulated biotic factors for osteochondral tissue regeneration

As mentioned above, the biotic factors, such as growth factors and cells, play important roles in osteochondral defect reconstruction. A variety of biphasic scaffolds encapsulate biotic factors through different strategies, such as individually loading one growth factor in one layer, *i.e.,* TGF-β1 in cartilage layer [25] and BMP-2 in subchondral bone layer [59]. The cartilage- and osseous-related growth factors in scaffolds are demonstrated to promote the regeneration of cartilage or subchondral tissue [31].

The biphasic scaffolds have been designed to load two kinds of growth factors sumptuously in different layers. As a typical instance, Re’em reported that the chondroinductive TGF-β1 was loaded in one layer and osteoinductive BMP-4 was loaded in the second layer to promote human MSCs differentiation into two end-stage lineage tissues. The histologic results indicated that MSCs were able to sense biological cues spatially presented in the different layered hydrogels and respond by differentiating into appropriate cell lineages [2]. In addition, the segmented polyurethane/PLGA bilayer scaffold enveloping both TGF-β1 and BMP-2 demonstrated a consistently good control of release kinetics. Moreover, the implantation of bilayer scaffold created fibrocartilage after 2 weeks, and resulted in high-quality hyaline neocartilage at 24 weeks later [60]. The excellently repaired osteochondral tissues converted the bilayer systems with rational loading of growth factors into a promising candidate for future applications in osteochondral lesions.

Cells also have an important position in the design and fabrication of bioactive biphasic scaffolds. Similar to growth factors, cells, like chondrocytes, MSCs, and pre-differentiated MSCs, are seeded in scaffolds in various ways according to the different structures of scaffolds. The chondrocytes are always implanted into the cartilage layer [49, 59, 61–63]. MSCs can be loaded into one layer [55] or both layers [51, 64]. Although most of the cell-seeded scaffolds show positive results in the regeneration of osteochondral tissue [32, 65], several studies indicate that no significant correlation of the repair outcomes toward osteochondral defects with the seeded cells [49, 58].

**Figure 4.**
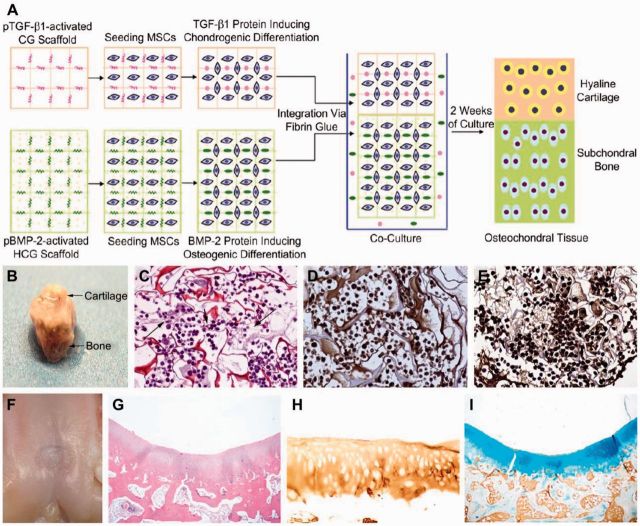
Diagrammatic representation of construction procedure of bilayer gene-activated composite osteochondral graft along with MSCs loaded into TGF-β1-activated CG scaffold layer and BMP-2-activated HCG scaffold layer (A). Macroscopic observation (B), H&E staining (C), and immunohistochemical staining of Col II (D) and Col I (E) of bilayer gene-activated osteochondral graft after 2 weeks of culture *in vitro* (× 200). Macrophotography of osteochondral defect repair *in vivo* (F), histological analysis by H&E staining (G), immunohistochemical staining of Col II (H), and immunohistochemical staining of Col I and Alcian blue staining for hyaline cartilage (I) after implanting bilayer gene-activated composite osteochondral scaffold incubated for 2 weeks *in vitro* for 12 weeks. (Reprinted with permission from Ref. [44])

The most ideal biphasic scaffold is composed of two growth factors of chondrogenic and osteogenic with host cells loaded in separated layers. As reported by Chen *et al.* [44], a bilayer gene-activated osteochondral scaffold was formulated consisting of plasmid TGF-β1)-activated chitosan-gelatin (CG) scaffold for chondrogenic layer and plasmid BMP-2 (pBMP-2)-activated HA/chitosan-gelatin (HCG) scaffold for osteogenic layer (Fig. 4). As shown in Fig. 4, the results showed that the spatially controlled and localized gene delivery system in the bilayer integrated scaffolds could induce MSCs in different layers to differentiate into chondrocytes and osteoblasts *in vitro*, respectively, and simultaneously support the articular cartilage and subchondral bone regeneration in the rabbit knee osteochondral defect model. The fascinating outcomes indicated that the multi-tissue regeneration through the combination of biomimetic and multi-phasic scaffolds and multi-lineage differentiation of a single stem cells represented a promising strategy for facilitating the development of complex tissue or organ systems.

## CLINICAL APPLICATIONS OF BIOINSPIRED SCAFFOLDS

Many great progresses have achieved for osteochondral reconstruction by biphasic scaffolds *in vitro* or preclinical studies *in vivo*. Moreover, there have been two novel bilayer scaffolds approved in clinical usage, that is, MaioRegen® (Fin-Ceramica Faenza SpA, Faenza, Italy) [66–73] and TruFit^TM^ Plug (Smith & Nephew, Andover, MA) [74–76].

MaioRegen® is a monolithic and bilayer scaffold mimicking the whole osteochondral unit. The superficial layer consists of Col I and resembles the cartilaginous tissue, whereas the lower layer consists mostly of magnesium-enriched hydroxyapatite (Mg-HA) simulating the subchondral bone structure [77]. The intermediate layer composed of col and Mg-HA reproduces the tide-mark. TruFit^TM^ plug is a bilayer cylindrical plug composed of PLGA fiber and calcium sulfate (CaSO_4_), and the reported clinical outcomes are controversial [78].

MaioRegen® has been systematically evaluated in patients. The international knee documentation committee (IKDC) subjective score of the suffer knee was improved significantly, the same positive trend was confirmed by the visual analogue scale and Tegner scores at 24 months after implantation [68, 72]. The results showed it was a promising strategy for OCD treatment, although abnormal magnetic resonance imaging findings were presented [72]. Another study has been carried out in 11 patients for the treatment of tibial plateau lesions. After 2 years follow-up, results showed a promising clinical outcome [70]. Recently, Christensen *et al.* [73] reported the analogous results of bilayer MaioRegen® for osteochondral defect repair after 1–3 years clinical and radiological follow-up. The results showed incomplete cartilage repair and poor subchondral bone repair at 1 and 2.5 years follow-up. But the clinical scores were significantly improved. The author showed great concerns about the biological potential repair *via* MaioRegen® scaffold.

Another commercial bilayer scaffold, *i.e.,* TruFit^TM^ Plug, undergoes a systematic-analysis of clinical application results. The conclusions showed there were no data available that support the superiority or equality of TruFit^TM^ Plug compared with conservative treatments or mosaicplasty/microfracture [76]. The randomized controlled clinical trials comparing with biphasic scaffolds through an established treatment method are needed before further clinical use can be supported. As for clinical application, MaioRegen® was implanted more than TruFit^TM^ Plug, as it was approved several years earlier.

## CONCLUSIONS AND FORECAST

As aforementioned, osteochondral defect repair is still a great challenge for both tissue engineers and orthopedic surgeons. Fortunately, some inspiring progresses have been made over the past decade toward osteochondral defect models. Even in clinic, several biphasic scaffolds have been approved for osteochondral defect reconstruction. Up to now, most of the biphasic scaffolds are made from natural and synthetic polymers, other high stiffness materials or their complexes, most of which claim acceptable results, while the ambiguous conclusions have also been reported [73].

Although growth factors and cells play important roles in tissue engineering, the same good functional results are obtained without them in many cases [69, 72]. Especially for commercial biomimetic scaffolds, it is hard to be restored and transported owing to the instability of growth factors, so they usually are growth factor free, *e.g.,* bilayer MaioRegen®. The ambiguous conclusions in both animal experiments and localized clinical trials reveal that the further studies are still required. Furthermore, accompanying with the developments of printing precision and materials technology, 3D printing technology provides a possible way to fabricate complex spatial structural scaffolds.

In one word, a promising scaffold will not only integrate both cartilage and subchondral bone to achieve a structural reconstruction, but also provide a satisfied long-term time fellow-up clinical outcome. Overall, the successful application of biomimetic biphasic scaffolds for osteochondral defect repair still needs further exploration.
